# Dealing with the adaptive immune system during *de novo* evolution of genes from intergenic sequences

**DOI:** 10.1186/s12862-018-1232-z

**Published:** 2018-08-03

**Authors:** Cemalettin Bekpen, Chen Xie, Diethard Tautz

**Affiliations:** Max-Planck Institute for Evolutionary Biology, August-Thienemannstr. 2, 24306 Plön, Germany

**Keywords:** Transcriptome, *de novo* genes, Adaptive immune system, *Aire*, Mouse populations

## Abstract

**Background:**

The adaptive immune system of vertebrates has an extraordinary potential to sense and neutralize foreign antigens entering the body. *De novo* evolution of genes implies that the genome itself expresses novel antigens from intergenic sequences which could cause a problem with this immune system. Peptides from these novel proteins could be presented by the major histocompatibility complex (MHC) receptors to the cell surface and would be recognized as foreign. The respective cells would then be attacked and destroyed, or would cause inflammatory responses. Hence, *de novo* expressed peptides have to be introduced to the immune system as being self-peptides to avoid such autoimmune reactions. The regulation of the distinction between self and non-self starts during embryonic development, but continues late into adulthood. It is mostly mediated by specialized cells in the thymus, but can also be conveyed in peripheral tissues, such as the lymph nodes and the spleen. The self-antigens need to be exposed to the reactive T-cells, which requires the expression of the genes in the respective tissues. Since the initial activation of a promotor for new intergenic transcription of a *de novo* gene could occur in any tissue, we should expect that the evolutionary establishment of a *de novo* gene in animals with an adaptive immune system should also involve expression in at least one of the tissues that confer self-recognition.

**Results:**

We have studied this question by analyzing the transcriptomes of multiple tissues from young mice in three closely related natural populations of the house mouse (*M. m. domesticus*). We find that new intergenic transcription occurs indeed mostly in only a single tissue. When a second tissue becomes involved, thymus and spleen are significantly overrepresented.

**Conclusions:**

We conclude that the inclusion of *de novo* transcripts in the processes for the induction of self-tolerance is indeed an important step in the evolution of functional *de novo* genes in vertebrates.

**Electronic supplementary material:**

The online version of this article (10.1186/s12862-018-1232-z) contains supplementary material, which is available to authorized users.

## Background

The adaptive immune system has evolved to provide receptors that can sense and eventually neutralize any foreign antigen entering the body. But this requires the system also to make a clear distinction between self and non-self antigens. This involves the maturation and selection of T-cells mostly in the thymus, but also other tissues, especially the lymph nodes and the spleen [1–5]. Medullary thymic epithelial (mTECs) and dendritic cells have an important function in this process as they express a large number of tissue-specific self-antigens, which are presented to developing T-cells [6–9]. This leads to the induction of tolerance by clonal deletion and functional inactivation of self-reactive T-cells. While this process starts during fetal development, T-cell maturation continues throughout life and is also particularly active in young adults [2, 6]. Transcription of the self-antigens to be exposed to the T-cells depends on the autoimmune regulator (AIRE) transcription factor [10, 11]. Targeted deletion of *Aire* causes a decreased expression of self-antigens in the thymus correlated with a development of autoimmunity [12]. Mutations in the *Aire* gene in humans cause the Autoimmune-Polyendokrinopathie-Candidiasis-Ektodermaldystrophie-Syndrome Type I (APECED) disease which is a syndrome characterized by the presence of autoantibodies that are specific for multiple self-antigens [13, 14] and *Aire* deficient mice develop multiple features of the APECED phenotype [15]. AIRE is not only expressed in the thymus, but also in extrathymic cells, including the spleen [5].

The phenomenon of *de novo* evolution of genes from intergenic sequences has by now been well documented [16–19]. The first stage in *de novo* evolution occurs when an intergenic DNA region comes under a new regulatory control to produce a distinct processed RNA transcript. Deep transcriptome sequencing has shown that much of the intergenic DNA is in fact transcribed [20, 21]. Studies between phylogenetically closely related populations, subspecies and species of house mice have shown that these intergenic transcripts show a fast turnover, i.e. are easily gained or lost within short periods, such that virtually the whole genome is “scanned” within a 10Myr time span [22]. Many of these new RNAs include an open reading frame and are translated [23–25], thus producing a completely new protein sequence. Such proteins can assume a functional role and could become true genes. But when such a new protein is presented by the MHC system to the cell surface, it would be recognized by the immune system as a foreign antigen thus leading to a destruction of the respective cells or cause inflammations. Hence, at least in animals with an adaptive immune system, one could predict that the *de novo* evolution of genes should be accompanied by an expression in the thymus and/or spleen to ensure the necessary self-tolerance.

We test this prediction here by studying the transcriptomes of different organs and the dynamics of new transcript emergence in three recently separated populations of the Western house mouse (*M. m. domesticus*). One represents an ancestral population from Western Iran (IRA), which is the source of the populations that have migrated towards Western Europe, starting probably less than 9000 years ago. The other two populations come from France (FRA) and Germany (GER) which split about 3000 years ago [26, 27]. We have previously shown that these populations harbor many new lineage-specific transcripts in brain, liver and testis [22]. We assess here a larger set of tissues, including new transcriptome sequences from the thymus.

We find that the largest number of different transcripts occurs in the testis and the brain, but these are immune privileged tissues, where auto-immunity would not play a role. With respect to transcript emergence across tissues, we find that *de novo* transcripts that are initially expressed in the thymus or the spleen have a much higher probability to become also expressed in other tissues and thus eventually to become functional. We conclude that the necessity for assuring self-tolerance is indeed an important factor in the emergence of *de novo* genes in species with an adaptive immune system.

## Results

Transcriptome data for the focal populations (IRA, GER, FRA) were previously generated for ten tissues (brain, gut, heart, kidney, liver, lung, muscle, spleen, testis and thyroid) [28]. To gain insight into the role of self-tolerance during the birth of new genes, we generated additional transcriptomes from the thymus of young adults for each of these populations. For the first set of analyses, we use averages of transcript numbers across the three populations since the differences between them are small.

**Fig. 1:**
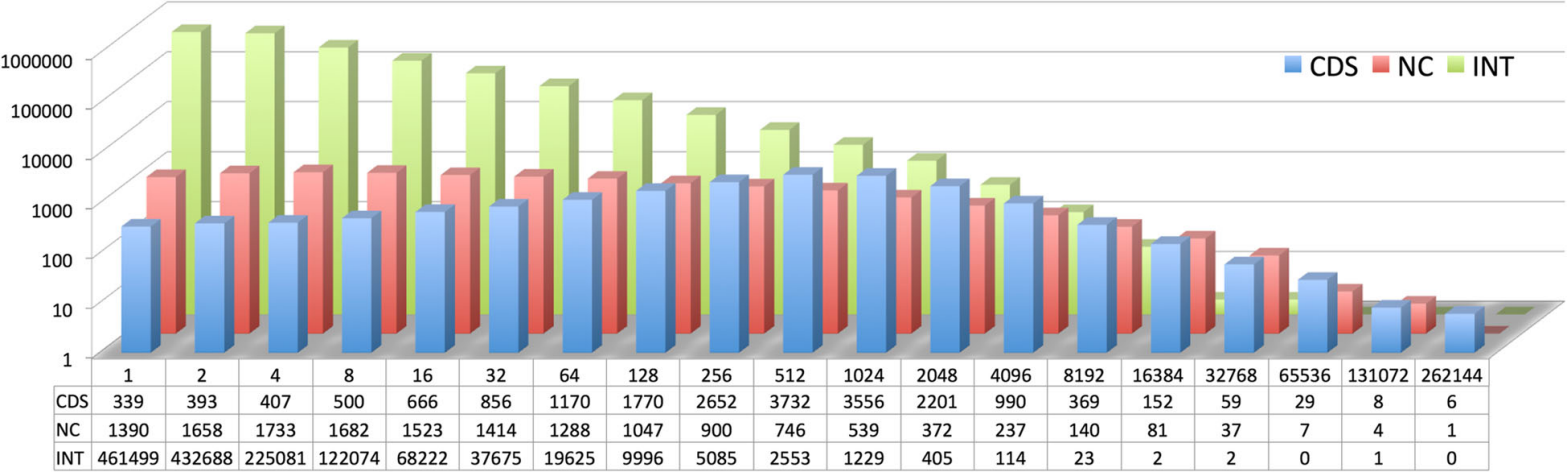
Distribution of transcript abundances. The data represent the sum of all normalized reads across all tissues and averages for the three populations. The X-axis shows bins of abundance in a logarithmic scale (bin number annotation: 1 = one read, 2 = > 1 to < 4 reads, 4 = > 3 to < 8 reads etc.) The Y-axis shows numbers of different transcripts that fall into the respective bin (log10 scale). The actual numbers are provided at the bottom. The three highest expression classes are not plotted because they include only 4 transcripts in total. CDS = annotated coding transcripts (blue), NC = annotated non-coding transcripts (red), INT = intergenic transcripts (green)

We distinguish three classes of transcripts for all comparisons: (1) annotated coding genes (CDS), (2) annotated non-coding genes including pseudogenes (NC) and (3) non-annotated intergenic transcripts (INT). The latter are the main candidates for *de novo* genes. Some *de novo* genes would also be expected to fall into one of the first two classes (see e.g. [23]), but we did not specifically correct for this. For CDS and NC, we counted the reads that match with the respective annotated gene models. In the absence of gene models for INT, we counted the overlap of transcripts within 200 bp windows across the whole genome, excluding the windows that are covered by annotated genes. Figure 1 provides an overview of the transcript abundances in each of these classes across the whole dataset.

The INT class has by far the largest number of different transcripts. However, these numbers are somewhat inflated compared to CDS and NC, since any given intergenic transcript would normally cover more than one window. Note that this makes the absolute numbers not directly comparable between CDS and NC on the one hand and INT on the other, since the former represent gene models, the latter are only numbers of 200 bp windows overlapping with a read. However, given that INT transcripts tend to be short, any full transcript is not expected to cover more than 3–5 windows. Most of the intergenic transcripts occur only at a low expression level, represented by only a few reads in the dataset (Fig. 1). In the following, we use a cutoff of at least eight reads (calculated as sum across the four individuals from each population - see methods) to call a transcript present in a given tissue and population. However, this cutoff is more or less arbitrary and the general patterns we describe below are not much affected by the exact cutoff used (we tested cutoff levels between 4 to 16).

### Tissue expression

**Fig. 2:**
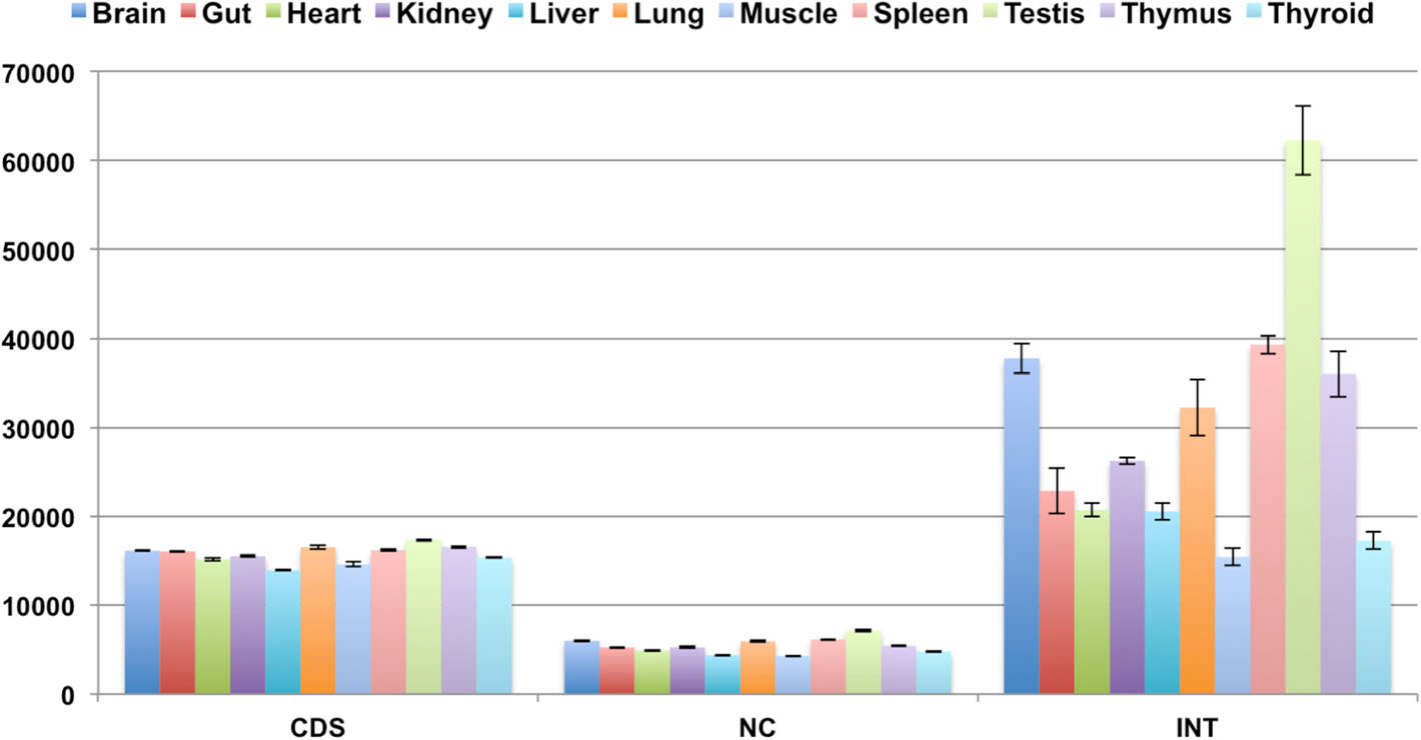
Numbers of transcripts in tissues. Numbers of transcripts found in each sampled tissue for the three classes CDS (annotated coding transcripts), NC (annotated non-coding transcripts) and INT (non-annotated intragenic transcripts). Averages were taken across all three populations, the error bars represent the standard error of the mean. Additional file 1 includes the corresponding data for this figure

Comparisons between the tissues show that testis expresses the relatively largest number of different transcripts in each of the three classes, followed by thymus, lung and spleen for CDS, spleen, brain and lung for NC and spleen, brain and thymus for INT (Fig. 2 and Additional file 1).

### Transcript sharing between tissues

The analysis of transcript sharing between tissues should allow to assess whether the emergence of a new transcript would occur initially in a single tissue and that broader expression in more tissues develops only later. We explored this question in several ways.

**Fig. 3:**
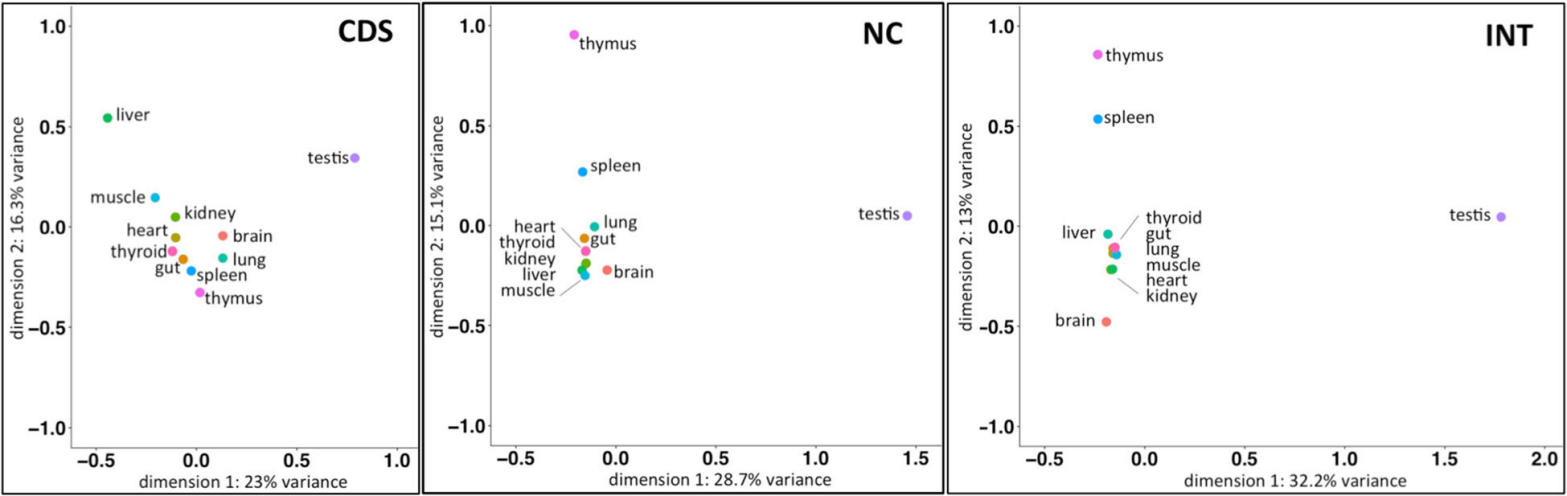
MCA analysis of tissues for the three expression classes. The first two dimensions are shown in each case with the % variance explained by them. The data represent the transcriptomes of the IRA population. The overall picture looks very similar for the GER and FRA populations (Additional file 2). CDS = annotated coding transcripts, NC = annotated non-coding transcripts, INT = intergenic transcripts

First we used a multiple correspondence analysis (MCA) [29] to assess overall similarities and differences of transcriptome sharing of the three classes between the tissues. For this, we generated a data matrix with four categories for each gene in each tissue, namely (1) no expression, (2) expression in only one tissue, (3) expression in two tissues and (4) expression in more than two tissues. We find for CDS that all tissues are distributed along the first two dimensions, with testis and liver as outliers (Fig. 3 and Additional file 2). For NC and INT, the patterns are different with respect to the outliers. Here we find that thymus and spleen form the outliers, together with testis (Fig. 3). This suggests that the average transcriptome sharing patterns are indeed rather distinct for these tissues in these two transcript classes, implying especially for thymus and spleen an additional expression mechanism that distinguishes them from the other tissues.

**Fig. 4:**
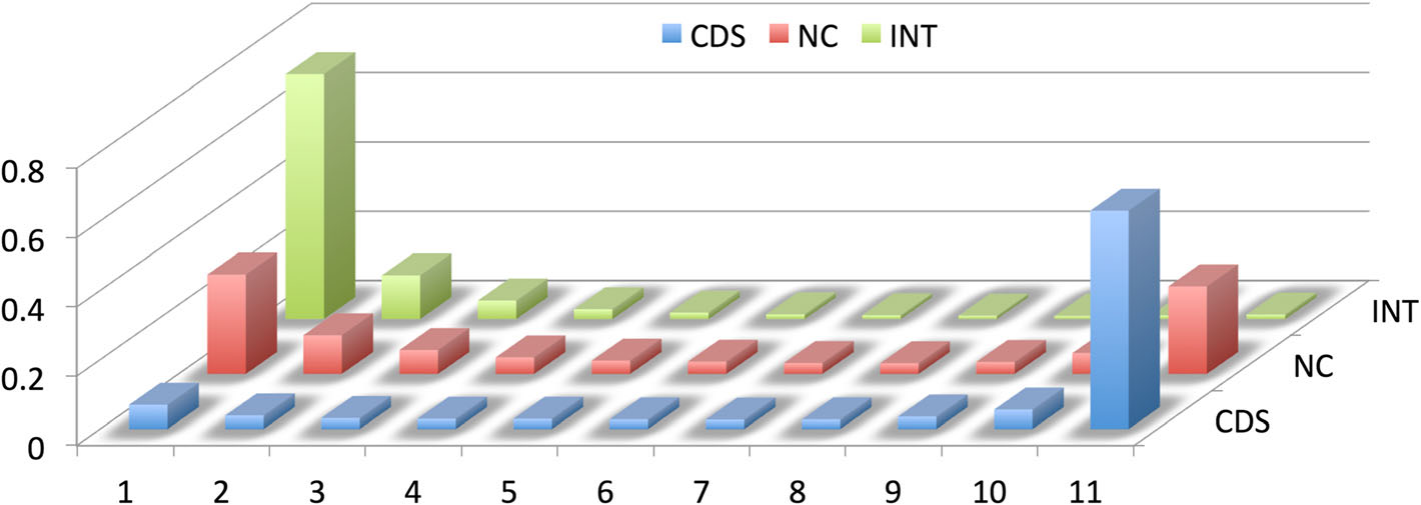
Transcript sharing across tissues. Fraction of transcripts for the three classes shared between numbers of tissues at the cutoff level of minimally eight reads. X-Axis: 1 means occurrence in a single tissue only, 11 means occurrence in all sampled tissues. Y-Axis represents the fraction of the total number of transcripts in each class. Numbers are averaged across all three populations. CDS = annotated coding transcripts (blue), NC = annotated non-coding transcripts (red), INT = intergenic transcripts (green). Additional file 3 includes the corresponding data for this figure

Second we asked whether INT transcripts are more likely to be expressed in a single tissue only. For this we analyzed whether a given transcript occurs in only one, two, three etc., up to all eleven tissues in the study. We find indeed rather contrasting patterns for the three classes of transcripts. While most CDS are shared between all tissues (at the minimum cutoff level of eight reads), INT is mostly specific to a single tissue only, while NC transcripts have both, many transcripts in single tissues, as well as many across all tissues (Fig. 4 and Additional file 3). This pattern suggests that intergenic transcription is at least initially mostly biased towards single tissues and is expected to reach higher expression levels across more tissues when the transcripts turn into functional genes.

**Fig. 5:**
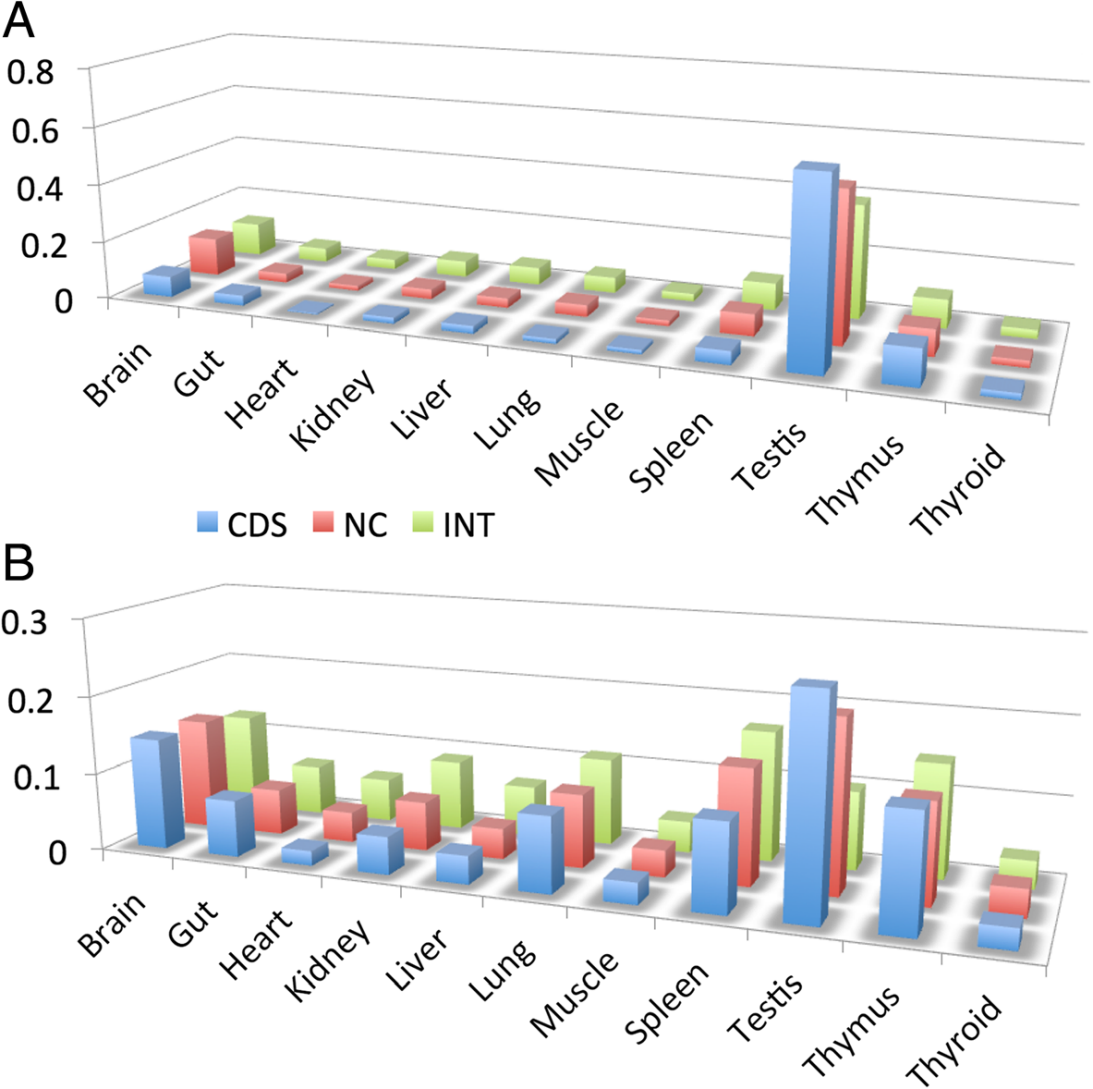
Tissue specific and shared transcripts across tissues. **a** Transcripts occurring only in single tissues represented as fractions for each class. **b** Transcripts occurring in two tissues represented as fractions for each class. Note that the second tissue can be any of the other tissues. CDS = annotated coding transcripts (blue), NC = annotated non-coding transcripts (red), INT = intergenic transcripts (green). Additional file 4 includes the corresponding data for this figure

Third, we asked which tissue expresses the largest fraction of tissue specific transcripts. We find that this is the testis with 40–65% of all transcripts in all three classes being expressed only in testis (Fig. 5a and Additional file 4). However, for the question of whether a testis expressed transcript becomes secondarily shared with another tissue, one has to ask which tissue expresses the largest fraction of transcripts that are shared between two tissues. Here we find that spleen and thymus show a much larger shared fraction than the tissue specific fraction (Fig. 5b). This is particularly clear for the INT class of transcripts, which has a higher fraction and higher numbers of shared transcripts compared to testis. The patterns are very similar when one considers 3 or more shared tissues (full data in Additional file 5).

### Turnover of transcripts

All the above analyses are based on the average number of transcripts per tissue between the three populations. To obtain an insight into how often a transcript expressed in one tissue gains a second expression in another tissue, we used the resolution that we get from analyzing the three populations separately. We restricted this analysis to the INT transcripts, since we found previously that there is only little turnover for CDS and NC in these very recently separated populations [22].

Using the IRA population as the phylogenetic stem group, we can ask which tissue shows the most gains of expression in other tissues in either one of the derived phylogenetic groups, FRA or GER. Note that any gain in one of the two derived groups could potentially be interpreted as a loss in both the stem group, as well as the other derived group. However, given that only a small percentage of transcripts show changes (see below) it is more parsimonious to assume one gain instead of two independent losses of the same transcript.

**Fig. 6:**
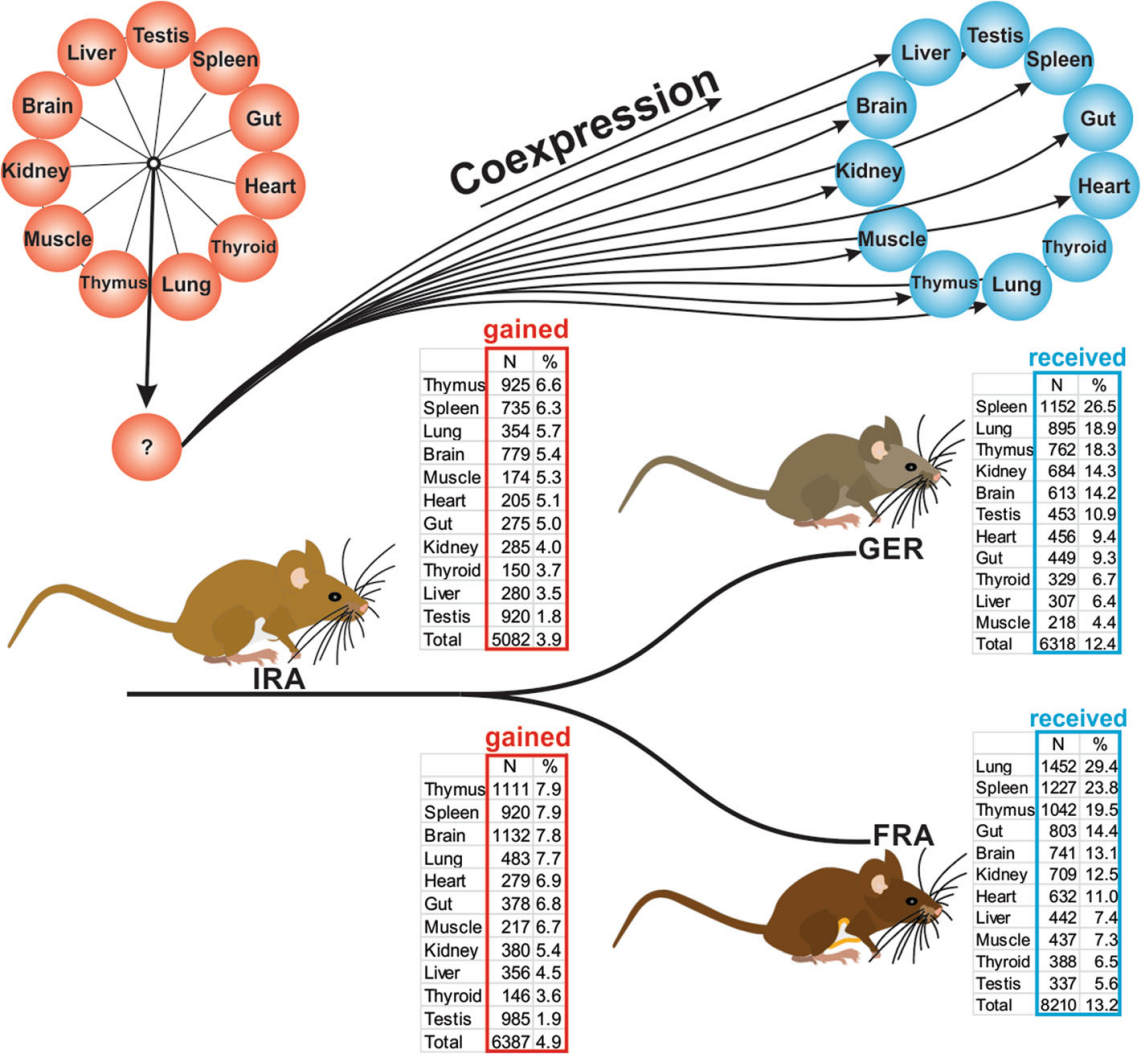
Gains of additional expression in other tissues in a phylogenetic context. The population from IRA represents the stem population for GER and FRA, which have separated about 3000 years ago. The figure depicts the analysis where we ask two different questions for each of the tissue specific INT transcripts of the IRA population. First, we ask for each of the tissues in IRA whether they gained a new transcript in GER or FRA in any other tissue. These numbers and percentages are presented in the left tables, labeled as “gained” (the top table is for GER, the bottom table for FRA). Here we find that transcripts initially expressed only in thymus, spleen, lung and brain in IRA have the highest percentage of gained transcripts in any other tissue in GER or FRA. Second, we ask which of the tissues in GER or FRA receive the additional expressions. These are the tables to the right, labeled as “received” (the top table is for GER, the bottom table for FRA). Here we find that lung, spleen and thymus are the tissues that receive additional expression from any of the IRA tissues. The tables present absolute numbers and the percentages for each tissue, scaled according to the total number of INT transcripts found in the respective tissue (left) and the total number of gains (right). The ranking of the tissues is according to this percentage value

To obtain the respective numbers, we used all windows that express a transcript in only one tissue in the IRA population and asked which of them shows an expression in at least one additional tissue in either FRA or GER. We find a total of 129,087 windows with expression in only one tissue in IRA. Of these single tissue expression windows, 5082 show at least one additional tissue expression in GER and 6387 in FRA (Fig. 6, left table, full data in additional file 6), i.e. between 4 and 5% of transcripts show such a change within the 3000 years of separation between the populations.

The ranking of the tissues in terms of the highest number of gains in one of the two other populations is summarized in Fig. 6. The four tissues with the largest number of gains are thymus, testis, brain and spleen. However, given the differences in the number of transcribed windows, the absolute values of new gains are a bit misleading. When one calculates the percentage of gains, thymus, spleen, lung and brain are the tissues with the relatively most gains, while testis has the least (Fig. 6, left table).

One can also ask which of the tissues in GER and FRA gain the largest number of new expressions, i.e., when a gene is expressed in only one tissue in IRA and has gained at least one new expression in either GER or FRA, which is the tissue that receives most of these new expressions. The results are depicted in the right table of Fig. 6. Again we find that thymus and spleen are among the tissues that receive the relatively largest numbers of gains, together with lung.

**Fig. 7:**
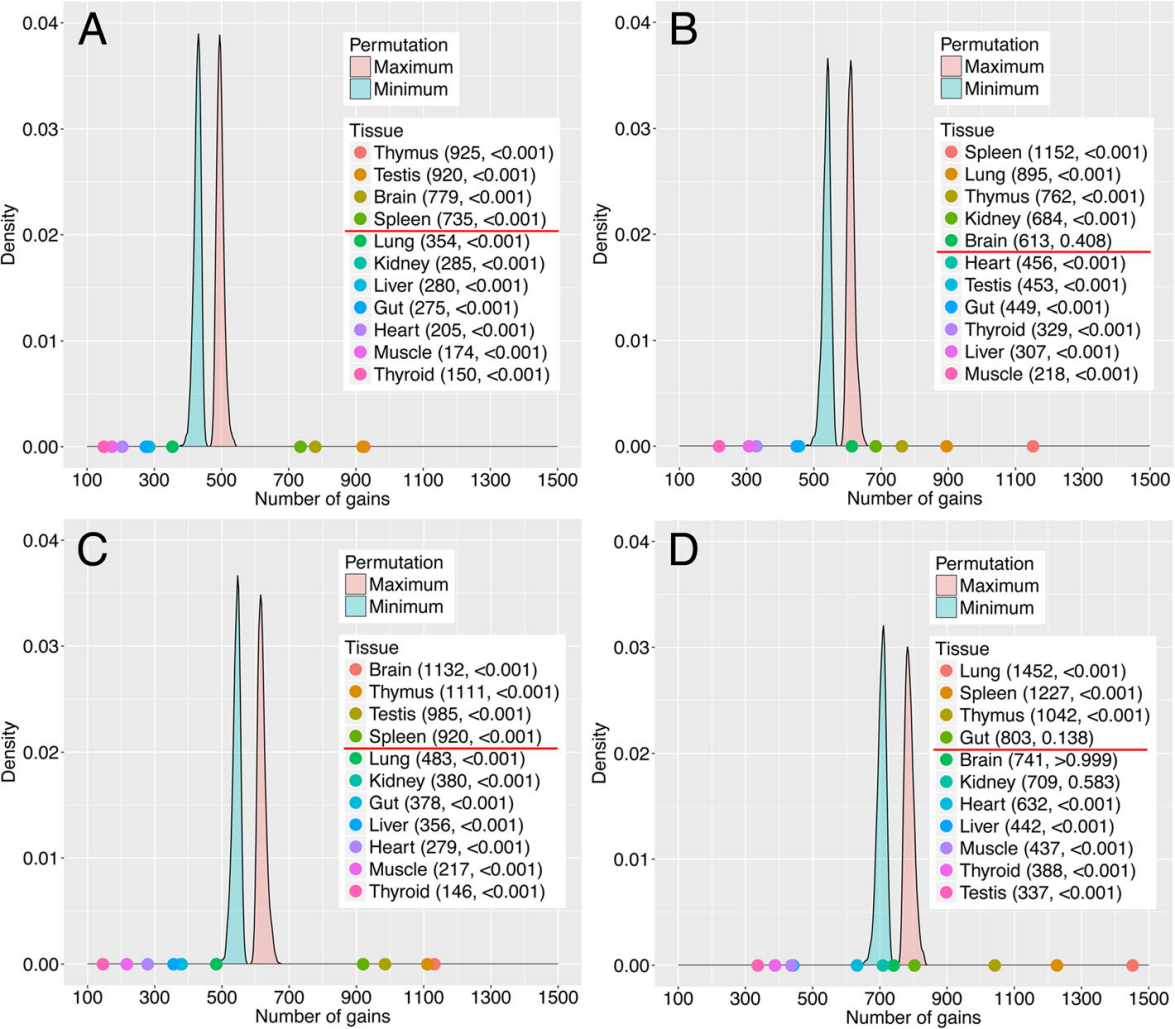
Simulation of random expectation of tissue sharing for INT transcripts. The distributions of minimum and maximum numbers for 1000 permutations of gains within the population context depicted in Fig. 6 are shown for the GER population at the top (**a** and **b**) and the FRA populations at the bottom (**c** and **d**). The left panels (**a** and **c**) represent the “gained” numbers, the right ones (**b** and **d**) the “received numbers” (see Fig. 6). The numbers of gains are plotted on the X-axis and the actual numbers for each tissue are provided in the boxes. *P*-values are added to the tables, the red line depicts the border above which there is an excess of gains

To test whether the excess of transcript sharing for particular tissues is different from random expectation, we have used a permutation based simulation to generate random distributions of the reads across tissues for each population. The results show that the number of gains for thymus and spleen are always significantly (*p* < 0.001) above the simulated random distribution (Fig. 7). Hence, we can reject the null hypothesis and conclude that the frequencies of different tissues to gain transcription are significantly different.

## Discussion

*De novo* emergence of new coding genes out of intergenic sequences should generate a problem for the adaptive immune system, since such new peptides would be recognized as foreign antigens and should lead to autoimmunity. This can be avoided when the new proteins are presented to the immune system during the development of self-tolerance. Thymus is considered to be the main organ where self-tolerance is induced through negative selection on T-cells that interact with the peptides in the body. However, there is also evidence that extrathymic cells in lymph nodes and the spleen can fulfill the same role [5]. Our data show that transcripts from intergenic sequences are indeed more often co-transcribed in these tissues than in any other tissue. This is most likely due to the *Aire* transcription factor, which is expressed in these two tissues and which is known to generate a promiscuous gene expression to allow the induction of self-tolerance [10, 11]. *Aire* acts by relieving the *Polycomb* induced silencing of transcription units that are normally part of the tissue-specific developmental program [9], although the exact mechanism is not yet clear. But one can assume that any *de novo* transcript that is part of a silenced chromatin region could become activated through *Aire* action, independent of a direct interaction of the locus with *Aire*. Much of the induction of immune tolerance happens already during fetal development, but is continued throughout life, particularly in young adults [2, 30]. Our tissue samples are from young adults rather than embryos. Hence, it is possible that the expression of new transcripts in the immune relevant tissues is even more pronounced at the embryonic stages, but the same principle is expected to operate at these stages.

*De novo* transcription of an intergenic sequence is expected to be caused by an initial random mutation that creates a new binding site for one of the transcription factors occurring in the respective cell. Hence, a new transcript could be “born” in any tissue. Interestingly, we find that INT transcripts are indeed often initially restricted to single or few tissues only, while long established genes tend to be expressed in most tissues. But this pattern is partly due to the choice of using a specific cutoff level for calling a transcript expressed or not expressed. If there are many transcripts that are expressed around the cutoff level, sampling stochasticity may or may not include a transcript in a given tissue into the category expressed or not expressed. Hence, we have tested several of the above analyses also with different cutoff levels, but we saw only changes in absolute numbers of transcripts, not in general patterns of sharing between tissues. Conversely, for highly expressed genes, it might be sufficient to have some leakage of transcription in tissues where they would otherwise not be expected to be expressed, to count them as being present in this tissue at the low cutoff level applied. Hence, the apparent tissue specificity of the INT genes may partly be a reflection of overall expression level, or in other words, the evolutionary transition from a *de novo* transcript into a full gene may mostly entail the increase of its expression level.

Given our criterion of counting any transcribed window outside of annotated gene models as INT, we include also cases where an annotated gene became extended by a new 5`- or 3´-UTR extension. This can create the possibility to translate additional short open reading frames [31–33], which may also contribute to *de novo* gene evolution. Hence, we have not specifically corrected for such cases, but such transcripts would be expected to retain the expression characteristics of their parental gene, i.e. would more likely behave as the genes from the CDS fraction.

In case a completely new INT transcript is expressed at a sufficiently high level and happens to have an open reading frame that is translated, it may create the above discussed problem for autoimmunity. If it does not get introduced to the self-tolerance mechanisms, it would be expected to be negatively selected and lost from the population. But if it is recognized by the self-tolerance mechanism, it could remain as a transcript in the population, even if it is itself more or less neutral. This would explain the relative enrichment of cases of co-expression between thymus and/or spleen and any other tissue for *de novo* evolved transcripts.

These considerations apply only to tissues that are subject to immune cell surveillance. Testis expression, especially in the postmeiotic stage, is not subject to such a surveillance. In addition, postmeiotic expression in testis is also rather promiscuous [6, 34, 35]. Given that these would not be subject to the negative selection outlined above, this could explain why one can find the largest number of *de novo *intergenic transcripts in testis.

A similar consideration applies to the brain, which is also largely immune privileged, i.e. T-cells have no access to most brain regions. Brain is not known to have a mechanism for promiscuous expression, but the large number of different cell types and differentiation stages implies that it expresses many transcription factors that could initiate *de novo* transcription according to the above mechanism. Hence, this, in combination of a lack of negative selection against new proteins, can explain why we find also in the brain large numbers of *de novo* transcripts. But although testis and brain express a large number of new INT transcripts, their percentage sharing capacity with expression in other tissues is smaller than for thymus and spleen. This is most evident for testis, where we see a particularly large number of new transcripts in combination with a particularly small percentage shared. This implies that the out-of-testis hypothesis for the origination of new transcripts [34, 35] needs to be adjusted for this fact.

Interestingly, we find also that the lung expresses many INT transcripts and is a preferred target for transcript sharing. The lung is immunologically very active, since it includes the defense against airborne infectious agents, i.e. expression of novel antigens from INT transcripts should present a problem. Hence, this remains currently an unresolved issue.

We note that our focus here was on polyadenylated transcripts, but it is well known that there are many non-polyadenylated transcripts from intergenic regions, originating from enhancers [36]. Such RNAs might also become functional [37], although not through their translation products. Hence, they would not interfere with the adaptive immune system.

## Conclusion

**Fig. 8:**
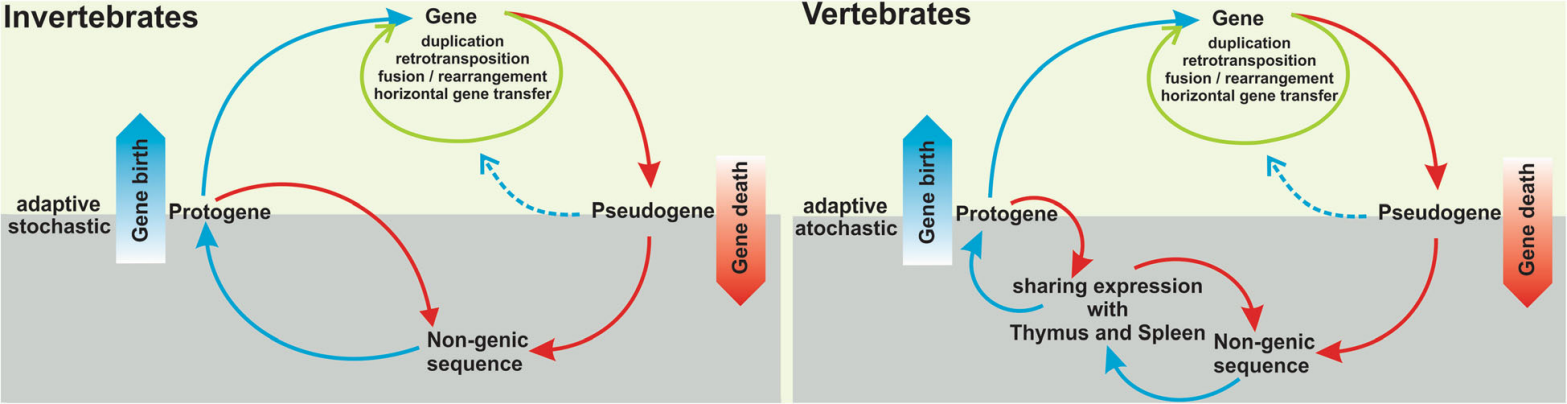
Scheme for the emergence of new genes. (Left) General scheme that was proposed by Neme and Tautz [38] that considers the emergence of *de novo* transcripts from non-genic sequences during a stochastic phase of genome evolution, where only random mutations allow the initial transcription of a given region. If this transcript is sufficiently stable, i.e. if it includes a poly-A tail and escapes other RNA destruction mechanisms in the cell, it would form a protogene that could become functional if adaptive conditions allow this. (Right) Extended version of the model, where the transcript would in addition have to be transcribed in thymus and/or spleen to avoid negative selection due to autoimmunity. This applies evidently only to transcripts with a translated open reading frame

Our finding of a role for the adaptive immune system in controlling the emergence of *de novo* genes implies that the dynamics of emergence are somewhat different between animals with and without an adaptive immune system. We have previously proposed a general scheme for new gene emergence that distinguishes an adaptive and a stochastic phase [38]. At least for vertebrates, we need to extend this by including the role of the adaptive immune system during the stochastic phase (Fig. 8).

## Methods

Transcriptome data for ten of the 11 organs used in this study were taken from [28]. For each population, we used the four individuals with the highest read coverage. The individual sample designations are provided in Additional file 7.

### Thymus samples

Thymus tissue was not included in the Harr et al. [28] transcriptome study. Hence, the tissues and transcriptomes were prepared for the present study. Mice of the three populations of *M. m. domesticus* (IRA, GER and FRA) were obtained from the breeding facility in Plön, where these animals are kept under outbreeding conditions [28]. Thymus tissues were prepared from four young individuals (between 4 and 8 weeks after birth) of each population. Note that while the process of distinction between self and non-self peptides starts already during embryonic development, it is still very active until about 8 weeks after birth [30]. CO_2_ asphyxiation and cervical dislocation was used to sacrifice the animals. The thymus is located just under the ribs, and looks like two thin white lobes overlying the heart tissue. After disconnecting the connective tissue surrounding the thymus, the pair of thymus lobes were carefully pulled and removed with curved serrated forceps without allowing bleeding. Note that the transcriptomes from the heart show also some *Aire* transcripts, but we conjecture that this is due to contamination with thymus tissue, which can easily happen when one does not pay particular attention on separating these tissues. The tissues were fresh frozen in liquid nitrogen immediately upon extraction and kept in liquid nitrogen to protect against RNA degradation until processing.

### RNA isolation and sequencing

Total RNA was isolated by using the RNeasy Microarray Tissue Mini Kit (Qiagen:73304) as recommended by the manufacturer. Transcriptome libraries were generated from the total RNA using the TruSeq RNA sample kit (Illumina). This selects for poly-adenylated mRNAs. Paired end sequencing (2 X 150 bp) was performed using the Illumina NextSeq 500 machine. Sequence reads were first checked for quality and trimmed by using Trimmomatic-0.30, using the options (ILLUMINACLIP:/opt/biosoftware/Trimmomatic/Trimmomatic-0.30/adapters/TruSeq2-PE.fa:2:30:10 CROP:101 LEADING:3 TRAILING:3 SLIDINGWINDOW:4:15 MINLEN:60).

### Mapping and normalization

The cleaned fastq files were mapped against the mm10 reference genome [39] with tophat2 [40] with default options. To estimate total countable reads from each tissue for normalization, reads from mapped, sorted, indexed Bam files were first counted to estimate unique mapped reads (not including multiple mapped reads) using featureCounts [41] with 200 bp non-overlapping window step size, with the option of --minOverlap 51.

By comparing the total counted mapped reads, each tissue was then normalized against the sample with the lowest number of mapped reads (AH kidney from Iran, 15,732,162 reads) by subsampling using the SAMtools software package. Given that four animals were used for each tissue, we combined these data, i.e. the analyses are based on a total of about 60 million reads per tissue. This implies that we are not studying here the biological variation of the individuals in each population, since our focus was on comparisons between populations. Normalized mapped reads were then again counted using featureCounts to extract the number of reads for the list of annotated protein coding genes (CDS) and non-coding transcripts (NC) (Ensembl vs. 83). Note that the NC fraction includes also all annotated processed and non-processed pseudogenes. All windows not falling into these annotations (note that the CDS and NC annotations include the intron regions) were considered as intergenic (INT) and final counting was performed against these windows. For several analyses we used the averages of the numbers determined singly for each population. Variances were generally low between the populations, i.e. the averaging does not influence the overall patterns. Regions corresponding to chrM, chrY, scaffolds, retro-genes (in cases where they are not annotated as pseudogenes - see above), repetitive LSU and SSU RNA were removed from the final analysis of INT regions, since they produce reads due to cross-mapping with their parental genes.

### Calculation of the gains per tissue in the population context

In order to determine the number of the gained additional tissue expression for either GER or FRA in INT regions, we first obtained all windows with single tissue expression (at the cutoff level of at least 8 reads summed across all four individuals) in IRA. By pairwise comparison of these single tissue expressed INT windows, we then determined the number of gained additional expression (one or more additional tissue) for each window in the GER and FRA populations (see Table “gained” in Fig. 6). In an alternative analysis, we asked which tissues received additional expression in GER or FRA from windows of single tissue expression in IRA. This includes cases with expression in multiple new tissues, but we counted among them only the cases that retained the original IRA expression tissue, plus a new expression in only GER or FRA (see Table “received” in Fig. 6).

### Multiple correspondence analysis

The R package FactoMineR (1.39) [29] was used for the MCA to reduce the n-dimension space and provide the first two converted dimensions which can explain the largest variances among the 11 tissues. The matrix used for the MCA was generated by assigning four categorical values for each gene in each tissue (summed across all four individuals in each population). The categories were: NE (no expression above the cutoff level of 8 reads), NS (no sharing, i.e. expression in only one tissue), SS (single sharing, i.e. expression in only two tissues) and MS (multiple sharing when expressed in three or more tissues).

### Simulation

To obtain an expected distribution for the frequency of transcript gains across tissues, we performed a permutation test based on the null hypothesis that the 11 tissues have equal probability to gain expression in every genomic region. In detail, in each permutation, for each genomic region which is expressed in at least one of the 11 tissues in one of the IRA, GER or FRA populations, we shuffled the tissue names, but retained the orthologous relation to obtain 11 “pseudo” tissues. Then we recalculated the number of gains of each “pseudo” tissue, and recorded the maximum and minimum numbers in each permutation. In total, we performed 1000 permutations and got two distributions of 1000 maximum numbers and 1000 minimum numbers separately.

## Additional files

Additional file 1: Source Table for Fig. 2. (XLSX 18 kb)

Additional file 2: MCA analysis of tissues for the three expression classes. The first two dimensions are shown in each case with the % variance explained by them. The data represent the transcriptomes of the GER and FRA populations (compare to Fig. 3 in the paper). CDS = annotated coding transcripts, NC = annotated non-coding transcripts, INT = intergenic transcripts. (PNG 345 kb)

Additional file 3: Source Table for Fig. 4. (XLSX 15 kb)

Additional file 4: Source Table for Fig. 5. (XLSX 18 kb)

Additional file 5: Full Table with numbers of transcripts per tissue for all populations and classes. (XLSX 18 kb)

Additional file 6: Gains of expression from single tissues in IRA to additional tissues in GER or FRA. The Table includes all INT windows that show an expression in a single tissue only (above the aggregate cut-off level of 8 reads) and that have gained at least one additional expression in either GER or FRA. (XLSX 5715 kb)

Additional file 7: Sample designations for transcriptome samples and read count overview. (XLSX 59 kb)

## Abbreviations

AIRE: Autoimmune regulator
APECED: Autoimmune-Polyendokrinopathie-Candidiasis-Ektodermaldystrophie-Syndrome Type I disease
CDS: Annotated coding genes
FRA: Mouse population from the Massif Central (France)
GER: Mouse population from the Cologne/Bonn area (Germany)
INT: Non-annotated intergenic transcripts
IRA: Mouse population from Ahvaz (Western Iran)
MCA: Multiple correspondence analysis
MHC: Major histocompatibility complex
mTEC: Medullary thymic epithelial cell
NC: Annotated non-coding genes including pseudogenes
