# Supplementary figures and images for: Dealing with the adaptive immune system during de novo evolution of genes from intergenic sequences
Source: BMC Evol Biol. 2018 Aug 3;18:121. doi: 10.1186/s12862-018-1232-z (PMC6091031; doi:10.1186/s12862-018-1232-z)

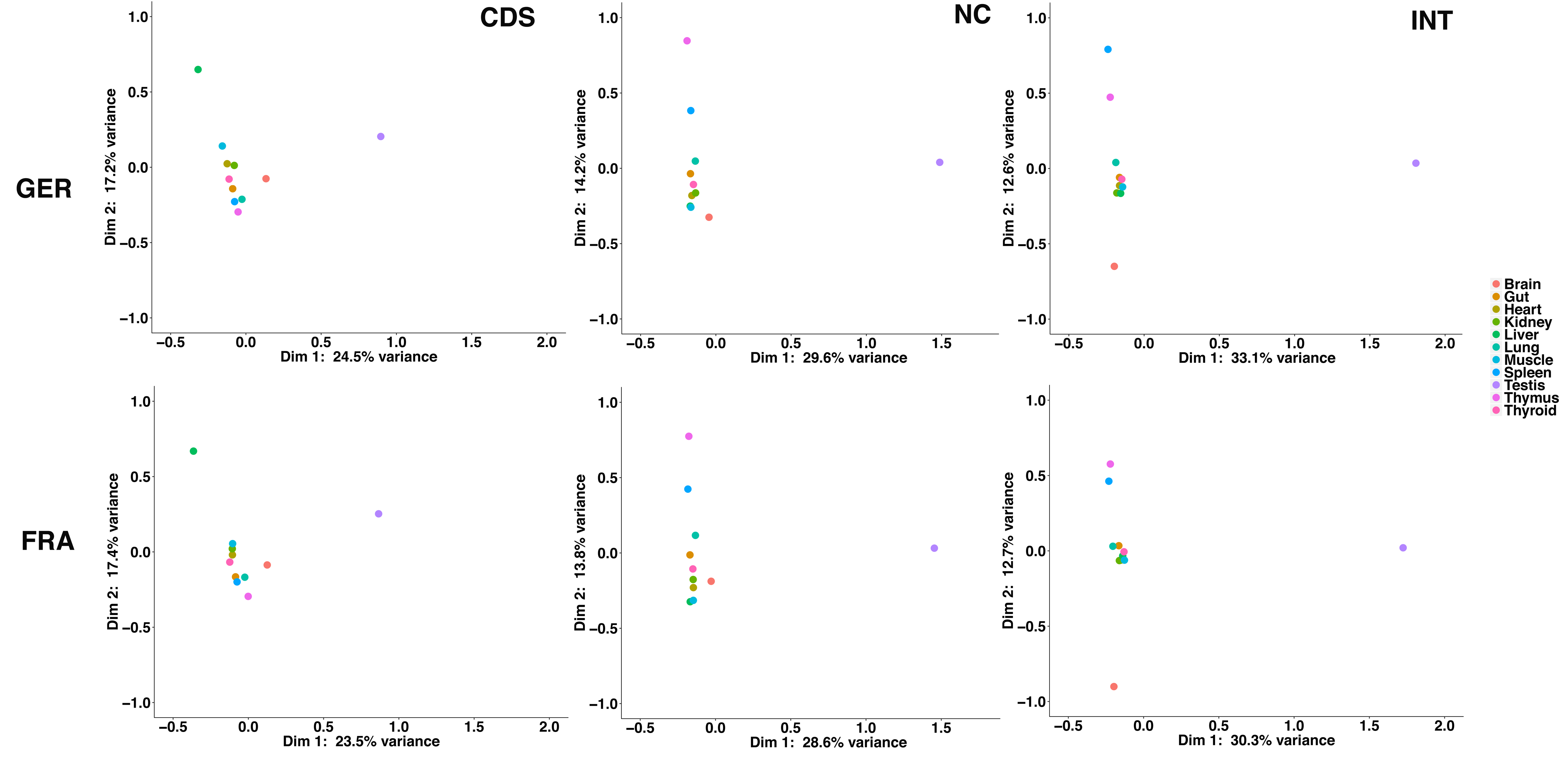

Supplement: Supplementary file 2 — MCA analysis of tissues for the three expression classes. The first two dimensions are shown in each case with the % variance explained by them. The data represent the transcriptomes of the GER and FRA populations (compare to Fig. 3 in the paper). CDS = annotated coding transcripts, NC = annotated non-coding transcripts, INT = intergenic transcripts. (PNG 345 kb) [file 12862_2018_1232_MOESM2_ESM.png]
